# Correlation between free fatty acids content and textural properties of Gouda cheese supplemented with denatured whey protein paste

**DOI:** 10.1007/s13197-022-05643-6

**Published:** 2022-12-07

**Authors:** Raid Ibrahem El-Metwally, Reham Kamal El-Menawy, Magdy Mohamed Ismail

**Affiliations:** Dairy Technology Research Department, Animal Production Research Institute, Agricultural Research Center, Dokki, Giza, Egypt

**Keywords:** Gouda cheese, Whey protein paste, Texture, Density, Free oil, Color, Fatty acids

## Abstract

In this study, the denatured whey protein paste (DWPP) was used to improve the texture characterizations of Gouda cheese. Five treatments of cheese were manufactured by adding 0, 1, 2, 3 and 4% of DWPP to cheese curd. Fortification of Gouda cheese with DWPP increased values of moisture, salt in moisture, water-soluble nitrogen/total nitrogen and non-protein nitrogen/total nitrogen whereas decreased values of density and free oil. The cheese contained DWPP was lighter and more yellowish compared to the control. The cheese samples contained 1 and 2% DWPP exhibited a significant increase in hardness, cohesiveness, springiness, gumminess and chewiness values while, the cheese samples that contained 3 and 4% DWPP exhibited a significant decrease. Adding DWPP to cheese lowered saturated fatty acids and raised unsaturated fatty acid (USFA) values which partially caused a lowering in cheese hardness at high levels of DWPP because of the low melting points of USFA. Based on these results, supplementation of Gouda cheese with 1 or 2% DWPP improved the texture properties.

## Introduction

The most important factors affecting cheese quality are aroma and flavor of cheese, texture, and appearance. Free fatty acids (FFA) emerged during lipolysis, especially short- and medium-chain FFA (C4:0–C8:0 and C10:0–C14:0, respectively), contribute together with the volatile compounds and the proteolysis products, directly to cheese flavor (Delgado et al. 2009). Long-chain FFA (> 14 carbon atoms) are considered to play a minor role in cheese flavor due to their high perception thresholds. On the other hand, the amount of free fatty acids in dairy products has potential effects not only on the taste and texture of dairy products but also on nutrition and health as antimicrobial agents (Çetinkaya1 and Öz 2018). Cheese, which is an important source of fat in human nutrition, contains a high level of various fatty acids. From a nutritional point of view, different types of cheese contain a high level of digestible fat. Its digestibility is in the range of 88–94% (Barać et al. 2018). Regarding Gouda cheese, triacylglycerols represent the main lipid fraction, followed by diacylglycerols, cholesterol, free fatty acids, and monoacylglycerols. Saturated fatty acids prevail in cheese fats, followed by monounsaturated fatty acids and polyunsaturated fatty acids, with palmitic, oleic, myristic, and stearic acids as the main ones (Semeniuc et al. 2022).

On the other side, the texture is a key factor in judging the quality of cheese and its functional attributes. Additionally, the texture is commonly used to recognize between different types of cheese. The texture of the cheese is a deciding element in public opinion and consumer preferences (Foegeding et al. 2003). The cheese texture is assessed either by sensory evaluation or instrumental estimations. The first method is time-consuming and needs intensive training for panelists. Therefore, for routine cheese texture analysis, the second method is often used (Kim et al. 2009). However, recently Finney et al (2001) showed the requirement for more appropriate practice to accomplish a high correlation between the instrument and sensory data for predicting cheese texture. In a Texture Profile Analysis (TPA), a food sample is compressed and decompressed twice, which simulates chewing food. The results obtained illustrate various texture observations that provide a strong correlation with those obtained by sensorial methods and are one of the most common tests for assessing the texture of cheese. Hardness, springiness, adhesiveness, and fracturability are the parameters most commonly used for TPA cheese (González et al. 2017).

Whey protein is an essential and economical protein source which contains all nine essential amino acids and branched amino acids. There are many benefits related to consumption of whey protein, like muscle building and loss of fat. It is full of immunoglobulins and can challenge cancer as an immune nutrient (Sangwan and Seth 2021). In recent decades, whey protein and its fractions have been used to improve the texture of cheese. Ismail et al (2011) used denatured whey protein to improve of low fat Mozzarella cheese characteristics. Mihulová et al (2013) studied effect of modified whey proteins on texture and sensory quality of processed cheese. Elbarbary and Saad (2019) used camel’s whey protein concentrate to improve of the quality of buffalo’s milk soft cheese. In general, many of the functional characteristics of whey proteins are found in their denature capability. Left denatured, their hydrophobic and hydrophilic regions allow them to be good emulsifiers and foaming agents as they can lie at an interface (Schmidt et al. 1984). In Egypt, large amounts of whey protein are made by heating and filtering the Ras cheese whey, usually in the winter. The resulting denatured whey protein paste is characterized by its cheap price and multiple uses. Although the use of whey protein in its various derivative forms (dried, concentrate, isolate, and hydrolysate) in cheese making has been extensively studied, no studies about the use of denatured whey protein paste in making Gouda cheese have been performed. Therefore, the main objective of this investigation is to study the effect of fortification with denatured whey protein past on the texture properties and free fatty acids pattern of Gouda cheese.

## Materials and methods

### Materials

Fresh cow’s milk was obtained from El-Serw Animal Production Research Station, Animal Production Research Institute, Egypt. Cheese starter consisted of *Lactococcus lactis subsp. Lactis* and *Lactobacillus helveticus* was obtained from Chr. Hansen’s Lab A/S Copenhagen, Denmark.

### Methods

#### Preparation of DWPP

Denatured whey protein paste was prepared as described by Ismail et al (2011). Ras cheese whey was skimmed and heated to 95 °C for 10 min., cooled and the flocculated denatured whey proteins were recovered by filtering through cheese cloth bags overnight. The precipitate was transferred to wooden frames and pressed for 2 h.

#### Manufacture of Gouda cheese supplemented with denatured whey protein

Fresh milk was pasteurized at 65 °C for 30 min, then cooled to 32 °C. After adding 0.02% CaCl_2_, milk was inoculated with culture (Direct Vat Set) at 2 g/100 L and incubated for 45 min. Then, rennet (Christian Hansen, Denmark) was added at 0.19 mL/kg of milk. After complete coagulation (~ 40 min.), curd was cut into 1.0 cm3 cubes, settled for 10 min, and then stirred at 32 °C for 20 min. A third of whey was drained, then an equal amount of warm water (~ 43 °C) was added to raise the temperature to 39 °C. Thereafter, more whey was drained, once again, warm water was added and at the same time, stirring of the curd continued for 30 min. Following draining all the whey, the denatured whey protein paste (0, 1, 2, 3 and 4%) was mixed with the curd before molding. After pressing overnight, the cheese was soaked in 19% NaCl solution for 8 h, dried in ripening room for one day, coated with plastic and matured in a cooling chamber (12 ± 0.5 °C, 85–90% relative humidity (RH)) for three months. Gouda cheese samples analyzed when fresh and after 1, 2 and 3 months of ripening period. Three replicates of each treatment were conducted.

#### Physicochemical analyses

Titratable acidity, moisture, fat, total nitrogen, water soluble nitrogen and non-protein nitrogen of samples were determined according to AOAC (2006) methods. The pH values of milk and DWPP samples were measured using a pH meter (Corning pH/ion analyzer 350, Corning, NY) after calibration with standard buffers (pH 4.0 and 7.0). Salt content was estimated using Volhard method according to Richardson (1985). Moisture in nonfat substance (MNFS) was calculated as moisture/(100 − fat) × 100.

#### Density and free oil measurements

Cheese density was determined by the water displacement technique according to Iezzi et al (2013). Cheese samples, after being diced into small particles (a cubic shape with 3–4-mm sides) were inserted into 50-mL volumetric flask, then distilled water at 20 °C was added up to the 50 mL mark. The flask and its content were weighed. The density of cheese was calculated as follows: *ρ*_*c*_ = *W*_c_/*V*_c_, where *W*_c_ is the cheese weight (kg) and *V*_c_ is the cheese volume (m^3^) found as 50 mL minus the ratio of weight of added water until the 50 mL mark and water density (1000 kg/m^3^).

Free oil content was measured according to Daia et al. (2019). Ground cheese (9 g for full-fat cheese) was put in a Babcock bottle and immersed in boiling water for 15 min to melt the cheese. Methanol diluted in distilled water (1:2, 20 mL at about 57.5 °C) was immediately added in bottle and then the bottle was centrifuged using a Babcock centrifuge at about 57.5 °C for 10 min. Free oil content of cheese was expressed as both percentage in cheese and percentage in cheese fat as: ${\text{Free}}\,{\text{Oil}}\,\left( {{\text{FO}}\,\% } \right)\, = \,{\text{reading}}\,{\text{of}}\,{\text{fat}}\,{\text{column}}/{\text{cheese}}\,{\text{weight}}.$

$${\text{Free}}\,{\text{Oil}}\,{\text{on}}\,{\text{fat}}\,{\text{basis}}\,\left( {{\text{FOFB}}} \right)\, = \,{\text{Free}}\,{\text{Oil}}\,\left( \% \right)/{\text{Total}}\,{\text{Fat}}\,(\% ).$$

#### Colour measurement

The colour of Gouda cheese was measured at the end of ripening period using Hunter Colorimeter Model D2s A-2 (Hunter Assoc. Lab. Inc. Va, USA) following the instruction of the manufacturer (Hunter colorimeter 1976).

#### Texture analyses

Texture Profile Analysis of cheese treatments ripened for three months was measured using a Texturometer model Mecmesin Emperor ^TM^Lite 1.17 (USA). Mechanical primary characteristics of hardness, springiness, gumminess and cohesiveness and also the secondary characteristic of chewiness (hardness x cohesiveness x springiness) were determined from the deformation Emperor ^TM^Lite Graph.

### Fatty acid analysis

#### Sample preparation

Identification and quantification of free fatty acids were performed according the method described by Jahreis et al (1997). Fat from 2 g cheese was extracted using 15 ml of Fokh's reagent (chloroform/ methanol = 2:1 (v/v)). The extracted lipids were filtered over anhydrous Na_2_S0_4_. Fatty acids methyl ester (FAMEs) was prepared by transesterification with potassium methylate. 0.5 ml potassium methylate (5% wt/wt in methanol) were added to the fat solution in the Pyrex@ tube. The tube was tightly capped, vortexed, and heated at 60 °C for 15 min in a drying cabinet. After cooling down 1.5 ml sulphuric acid (2% wt/wt in aq. dest.) was added and the tube was vortexed again. 1 µl from the clear organic phase was injected into the gas chromatograph.

### Gas chromatography (GC) analysis

Fatty acids were determined by gas–liquid chromatography where 1 µl of FAMEs was injected into GC–MS auto sampler (7890 A GC System Agilent) fitted with MSD detector, using ZB-5 fused silica capillary column. To identify the fatty acids, the fatty acids in mg g^−1^ total lipids were quantified in relation to the internal standard, methyl tricosanoate (23:0) from Sigma. Before transesterification, 1.00 mL of internal standard solution (1 mg mL-1) was added to all samples and the solvent was evaporated under N_2_ flow.

### Statistical analysis

Analysis of variance (ANOVA) was conducted using SPSS 17.0 program. Significant differences among samples were determined using Duncan’s multiple range test (*p* < 0.05).

## Results and discussion

### Physicochemical properties of milk and DWPP used in Gouda cheese manufacture

Findings of the physicochemical characteristics of milk and DWPP used in Gouda cheese preparation were outlined in Table 1. DWPP had high acidity and low pH values which may be due to an increase in whey acidity during Ras cheese making. The fat content of DEPP was very low (0.82%). This can be the result of skimming the whey. As expected, protein level of DWPP was high. As a result of adding salt in Ras cheese production, the salt content of DWPP increased. Generally, results of the chemical composition of DWPP obtained in this study located in the ranges cleared by Ismail et al (2011).

**Table 1:** Physicochemical properties of milk and DWPP preparation used in Gouda cheese manufacture

	Acidity %	pH values	TS %	Fat%	Total protein %	Salt %
Cow's milk	0.17	6.65	12.01	3.92	3.38	–
DWPP	0.26	5.38	25.27	0.82	15.86	7.34

### Some ripening indices of Gouda cheese

Table 2 displays outcomes of moisture, moisture in nonfat substance (MNFS), salt in moisture, fat/DM, TN/DM, WSN/TN and NPN/TN values of Gouda cheese during the ripening period. The moisture content was slightly higher in cheese fortified with DWPP comparing with control which may be due to the high water holding capacity of whey protein past. Lo and Bastian (1998) reported that the water-holding capacity of native whey proteins can be increased by heat treatments that partially denature these proteins. The cheese treatments manufactured with adding DWPP also had higher levels of all mentioned ripening indices. Moreover, the rates of increase in these indices during the ripening period were higher in DWPP cheese. These results revealed that more proteolysis occurred in DWPP cheese than control which can be considered as a ripening acceleration. In contrast to these results, Harper et al (1989) have found that denatured whey proteins had little effect on *α*_sl_-casein degradation but inhibited *β*-Casein proteolysis in Cheddar cheese slurries. Supporting our results, Guinee et al (1995) stated that high heat treatment of milk prior to ultrafiltration produces cheese with high degree of primary proteolysis as compared with UF cheese manufactured from milk heated at lower temperatures, which suggested that denatured whey proteins do not impede the hydrolysis of casein in cheese. Also, the increasing of ripening indices of Gouda cheese as a result of adding DWPP can be explained according to: (a) the presence of DWPP raised the production of pH 4.4 soluble N (Harper et al. 1989), (b) the stimulation impact of DWPP on cheese microorganisms. As it is known, the numbers of microorganisms present in the cheese have a very important and positive role in the ripening process, either directly through their metabolic activity or indirectly through the release of proteolytic and lipolytic enzymes into the cheese matrix as a result of the autolysis process after death. Fitzpatrick and O’Keeffe (2001) stated that fortification of whey permeate by whey protein hydrolysate had a beneficial influence on lactose utilization and *Lactobacillus helveticus* growth during fermentation.

**Table 2:** Effect of adding DWPP on moisture content and some ripening indices of Gouda cheese

Properties	Treatments	Ripening period (Month)				
		0	1	2	3	Means ± SD
Moisture %	I	41.57 ± 1.00	40.19 ± 1.00	39.20 ± 1.00	38.46 ± 1.00	39.85 ± 1.48^B^
	II	41.89 ± 1.00	40.50 ± 1.00	39.55 ± 1.03	38.80 ± 1.00	40.18 ± 1.48^B^
	III	42.16 ± 1.00	40.75 ± 1.00	39.78 ± 1.00	39.09 ± 1.00	40.45 ± 1.48^B^
	IV	42.38 ± 1.00	40.96 ± 1.00	40.00 ± 1.00	39.27 ± 1.00	40.65 ± 1.48^AB^
	V	42.54 ± 1.00	42.12 ± 1.00	41.16 ± 1.00	39.48 ± 1.00	41.33 ± 1.49^A^
	Means ± SD	42.11 ± 0.92^a^	40.90 ± 1.09^b^	39.93 ± 1.09^c^	39.02 ± 0.92^d^	
MNFS* %	I	58.42 ± 1.00	57.10 ± 1.00	56.01 ± 1.00	55.35 ± 1.00	56.72 ± 1.31^B^
	II	58.83 ± 1.05	57.48 ± 1.03	56.54 ± 1.02	55.77 ± 1.00	57.15 ± 1.33^B^
	III	59.15 ± 1.00	57.78 ± 1.00	56.80 ± 1.00	56.13 ± 1.03	57.46 ± 1.37^B^
	IV	59.41 ± 1.07	58.03 ± 1.00	57.07 ± 1.00	56.12 ± 1.04	57.66 ± 1.35^AB^
	V	59.65 ± 1.00	59.62 ± 1.00	58.66 ± 1.05	56.59 ± 1.00	58.63 ± 1.40^A^
	Means ± SD	59.09 ± 0.95^a^	58.00 ± 1.00^b^	57.02 ± 1.04^c^	55.99 ± 0.90^d^	
Salt in moisture %	I	5.46 ± 1.00	6.77 ± 1.00	7.88 ± 1.00	8.55 ± 1.00	7.16 ± 1.49^A^
	II	5.61 ± 1.00	7.04 ± 1.00	8.14 ± 1.00	8.76 ± 1.00	7.39 ± 1.51^A^
	III	5.83 ± 1.00	7.26 ± 1.00	8.32 ± 1.00	8.95 ± 1.00	7.59 ± 1.50^A^
	IV	6.01 ± 1.00	7.40 ± 1.00	8.48 ± 1.00	9.14 ± 1.00	7.76 ± 1.50^A^
	V	6.18 ± 1.00	7.41 ± 1.00	8.48 ± 1.00	9.35 ± 1.00	7.86 ± 1.56^A^
	Means ± SD	5.82 ± 0.89^c^	7.18 ± 0.88^b^	8.26 ± 0.88^a^	8.95 ± 0.89^a^	
Fat/DM %	I	49.36 ± 1.00	49.52 ± 1.00	49.36 ± 1.00	49.58 ± 1.00	49.45 ± 0.86^B^
	II	49.54 ± 1.00	49.66 ± 1.00	49.71 ± 1.00	49.72 ± 1.00	49.66 ± 0.86^AB^
	III	49.65 ± 1.00	49.76 ± 1.00	49.77 ± 1.00	49.84 ± 1.00	49.72 ± 0.86^AB^
	IV	49.74 ± 1.00	49.83 ± 1.00	49.85 ± 1.00	49.45 ± 1.00	49.76 ± 0.87^AB^
	V	49.74 ± 1.00	50.71 ± 1.00	50.69 ± 1.00	49.97 ± 1.00	50.28 ± 0.96^A^
	Means ± SD	49.60 ± 0.86^a^	49.71 ± 0.95^a^	49.88 ± 0.96^a^	49.89 ± 0.87^a^	
TN/DM %	I	5.87 ± 0.01	6.76 ± 0.01	6.87 ± 0.57	7.00 ± 0.01	6.71 ± 0.58^E^
	II	6.12 ± 0.01	6.95 ± 0.01	7.17 ± 0.01	7.19 ± 0.01	6.86 ± 0.46^D^
	III	6.28 ± 0.01	7.24 ± 0.01	7.46 ± 0.01	7.52 ± 0.01	7.13 ± 0.52^C^
	IV	6.53 ± 0.01	7.65 ± 0.01	7.90 ± 0.01	7.90 ± 0.01	7.49 ± 0.59^B^
	V	6.85 ± 0.01	7.90 ± 0.01	8.23 ± 0.01	8.16 ± 0.01	7.79 ± 0.58^A^
	Means ± SD	6.33 ± 0.35c	7.30 ± 0.44^b^	7.59 ± 0.48^a^	7.56 ± 0.45^a^	
WSN/TN %	I	9.89 ± 1.0	10.17 ± 1.0	10.44 ± 1.0	11.65 ± 1.0	10.54 ± 1.10^A^
	II	9.84 ± 1.0	10.55 ± 1.0	11.18 ± 1.0	12.23 ± 1.0	10.95 ± 1.25^A^
	III	9.84 ± 1.0	10.94 ± 1.0	11.31 ± 1.0	12.07 ± 1.0	11.04 ± 1.19^A^
	IV	9.97 ± 1.0	10.59 ± 1.0	11.79 ± 1.0	12.46 ± 1.0	11.20 ± 1.33^A^
	V	10.03 ± 1.0	10.86 ± 1.0	11.84 ± 1.0	12.37 ± 1.0	11.27 ± 1.27^A^
	Means ± SD	9.91 ± 0.85d	10.62 ± 0.89^c^	11.31 ± 0.99^b^	12.16 ± 0.89^a^	
NPN/TN %	I	3.96 ± 0.01	4.18 ± 0.01	4.63 ± 0.01	4.94 ± 0.01	4.43 ± 0.94^C^
	II	4.23 ± 0.01	4.50 ± 0.01	4.88 ± 0.01	5.34 ± 0.01	4.74 ± 0.44^CD^
	III	4.46 ± 0.01	4.63 ± 0.01	5.07 ± 0.01	5.55 ± 0.01	4.93 ± 0.44^AB^
	IV	4.66 ± 0.01	4.79 ± 0.01	5.21 ± 0.01	5.65 ± 0.06	5.07 ± 0.39^AB^
	V	4.76 ± 0.01	5.07 ± 0.01	5.17 ± 0.01	5.77 ± 0.01	5.19 ± 0.38^A^
	Means ± SD	4.41 ± 0.48^c^	4.63 ± 0.48^c^	4.99 ± 0.44^b^	5.44 ± 0.48^a^	

^a,^ ^b,^ ^c,^ ^d^letters indication to significant differences between the samples of Gouda cheese ±SD, ^A,^ ^B,^ ^C,^ ^D^letters indication to significant differences between storage period of Gouda cheese ±SD; Treatment I: Gouda cheese (control); Treatment II: Gouda cheese contained 1 % DWPP; Treatment III: Gouda cheese contained 2 % DWPP; Treatment IV: Gouda cheese contained 3 % DWPP; Treatment V: Gouda cheese contained 4 % DWPP

* MNFS, moisture in nonfat substance

### Density and free oil of Gouda cheese

As it is known, density is the mass of a material for each unit volume. The density values of Gouda cheese during ripening stage are reported in Table 3. The density results of four treatments contained DWPP were significantly lower (*P* < 0.05) than that of control in fresh cheese and throw ripening. Data of the previous table indicate an increase in the moisture content of the DWPP samples compared to control. As cleared in literature, the cheese moisture content has a negative significant correlation with density; therefore, this may be the reason for the lower density of DWPP treatments compared to control. Iezzi et al (2013) found a strong or moderate significant positive connection of density with ash, protein and total nonfat solids, and a moderate significant negative connection with moisture. No correlation of density with carbohydrates or fat was detected.

**Table 3:** Effect of adding DWPP on density (kg/m^3^) and free oil values of Gouda cheese

Properties	Treatments	Ripening period (Month)				
		0	1	2	3	Means ± SD
Density (kg/m^3^)	I	1027	1036	1042	1049	1038^A^
	II	1022	1030	1036	1041	1032^B^
	III	1019	1025	1032	1037	1028^C^
	IV	1012	1020	1025	1034	1022^D^
	V	1010	1015	1022	1028	1018^E^
	Means ± SD	1.018^d^	1025^c^	1031^b^	1037^a^	
Free Oil (%)	I	2.15	2.20	2.17	2.16	2.17^A^
	II	2.04	2.00	1.85	1.73	1.90^B^
	III	1.89	1.77	1.70	1.62	1.74^C^
	IV	1.80	1.59	1.38	1.24	1.50^D^
	V	1.49	1.41	1.23	1.09	1.30^E^
	Means ± SD	1.87^a^	1.79^b^	1.67^c^	1.57^d^	
FOFB* (%)	I	0.074	0.074	0.072	0.071	0.073^A^
	II	0.071	0.068	0.061	0.057	0.064^B^
	III	0.066	0.060	0.057	0.053	0.059^C^
	IV	0.063	0.054	0.053	0.041	0.053^D^
	V	0.052	0.048	0.041	0.036	0.044^E^
	Means ± SD	0.065^a^	0.061^b^	0.057^c^	0.052^d^	

^a,^ ^b,^ ^c,^ ^d^letters indication to significant differences between the samples of Gouda cheese ±SD, ^A,^ ^B,^ ^C,^ ^D^letters indication to significant differences between storage period of Gouda cheese ±SD; Treatment I: Gouda cheese (control); Treatment II: Gouda cheese contained 1 % DWPP; Treatment III: Gouda cheese contained 2 % DWPP; Treatment IV: Gouda cheese contained 3 % DWPP; Treatment V: Gouda cheese contained 4 % DWPP

* FOFB; Free Oil on Fat Basis

The free oil (FO) and free oil on fat basis (FOFB) values for the experimentally Gouda cheeses are outlined in Table 3. Cheese supported with DWPP had lower FO and FOFB levels than that of control during ripening. As previously mentioned, DWPP cheese contained high moisture and thus low total solids and fat. Therefore, one of the possible reasons for low FO and FOFB values is the low fat content of these treatments. These outcomes are in accordance with those reported by Daia et al. (2019) that free oil increased with fat contents in Mozzarella and Cheddar cheese. Everett and Olson (2003) showed that Cheddar cheese made from recombined milk containing fat globules coated with whey protein (*α*-lactalbumin, or *β*-lactoglobulin) had lower free oil values as compared with control. Another potential reason for the low FO and FOFB values of DWPP cheese is the high salt content. The salt effect can be explained by a synergistic correlation between proteolysis and protein swelling. At higher salt levels the degree of protein swelling is enhanced and this will exert pressure on fat globules inside the serum channels such that the globules are squeezed together within the protein matrix (McMahon et al. 1999). However, the free oil didn't increase because of swelling of the protein matrix but rather decreased at the higher salt levels. Everett et al (2004) showed that salt content had a significant impact on free oil formation. Lower salt levels led to higher values of free oil of Mozzarella cheese.

The values of FO and FOFB for DWPP samples decreased with ripening time whereas they remained unchanged for control cheese (Table 3). These findings are in close agreement with the results of Everett and Olson (2003) who detailed that free oil decreased during ripening period for whey protein (*α*-LA and *β*-LG)-coated fat globule Cheddar cheeses. The control cheeses either were unchanged or showed an increase in free oil during the 70 d period. Contrary to our results, Daia et al. (2019) showed that the free oil increased with storage time in Cheddar cheese.

### Color properties of Gouda cheese at the end of ripening period

The changes in the color characteristics of Gouda cheese as affected by adding DWPP were classified in Table 4. The results revealed that as the concentration of added DWPP increased from 1 to 4%, the L* and b* values also increased. This means that the cheese contained DWPP was lighter and more yellowish compared to control. The increased L* values for DWPP cheese may be due to the increased protein content (Table 2) which forms gel particles that cause more light scattering and increase the L* value of treatments (Aydemir and Dervisoglu 2010). Henriques et al (2013) reported that the lightness of the fresh cheese increased with increasing amounts of liquid whey protein concentrate added to fresh cheese. Also, Mileriene et al (2021) revealed that curd cheese contained thermo-coagulated acid whey protein (TAWP) was brighter in colour (L* value, 100.52) compared to control cheese (87.22). For all treatments, parameter a* (green–red axis) had negative values that refer to a slight green hue. The addition of DWPP led to less a* values for Gouda cheese.

**Table 4:** Color parameters (L*, a*, b*) of Gouda cheese at the end of ripening period

Treatments	Color parameters		
	L*	a*	b*
I	66.97	− 1.62	17.66
II	72.10	− 2.81	19.14
III	73.11	− 2.78	19.56
IV	77.23	− 3.01	19.60
V	77.51	− 3.07	19.81

L*: black–white axis; a*: green–red axis, b*: blue–yellow axis. Treatment I: Gouda cheese (control); Treatment II: Gouda cheese contained 1% DWPP; Treatment III: Gouda cheese contained 2% DWPP; Treatment IV: Gouda cheese contained 3% DWPP; Treatment V: Gouda cheese contained 4% DWPP

### Textural properties of Gouda cheese at the end of ripening period

The textural characteristics of Gouda cheese with regards to hardness, adhesiveness**,** cohesiveness, springiness, gumminess, and chewiness are shown in Table 5.

**Table 5:** Effect of adding DWPP on the textural properties of Gouda cheese at the end of ripening period

Treatments	Hardness (N)	Adhesiveness (MI)	Cohesiveness (ratio)	Springiness (MN)	Gumminess (N)	Chewiness (MJ)
I	27.35^ab^	1.73^c^	0.49^b^	4.35^ab^	12.30^b^	53.15^b^
II	28.66^a^	1.62^b^	0.53^ab^	4.98^a^	15.40^a^	61.52^a^
III	28.70^a^	1.63^b^	0.55^a^	4.95^a^	15.30^a^	61.45^a^
IV	23.78^c^	1.98^b^	0.44^c^	4.17^b^	10.70^c^	46.78^c^
V	19.52^d^	2.31^a^	0.36^d^	3.76^c^	6.50^d^	38.52^d^

^a,^ ^b,^ ^c,^ ^d^letters indication to significant differences between the samples of Gouda cheese ±SD; Treatment I: Gouda cheese (control); Treatment II: Gouda cheese contained 1 % DWPP; Treatment III: Gouda cheese contained 2 % DWPP; Treatment IV: Gouda cheese contained 3 % DWPP; Treatment V: Gouda cheese contained 4 % DWPP

N: Newton

The results of Table 5 indicate that the hardness of Gouda cheese was significantly impacted by adding of DWPP (*p* < 0.05), with values ranging from 19.52 to 27.35 N. Fortification of cheese with 1 and 2% DWPP (samples II and III respectively) had a positive effect on hardness, increasing their values. However, with the increase in the added concentrations of DWPP (3 and 4%, samples IV and V respectively), the effect was quite negative, as the hardness values significantly lowered. These results may be because of the high retention of moisture in Gouda cheese supplemented with high levels of DWPP. These data are partly consistent with those obtained by Hanafy et al (2016) who cleared that fresh soft cheese contained 2 or 4% whey protein concentrate (WPC) had higher hardness values than control, but incorporation of 6% WPC significantly decreased cheese hardness. In the same trend, Tashakori et al (2013) stated that using WPC increased the softness of Feta cheese. When comparing between the results of chemical composition and hardness of Gouda cheese, it becomes clear that the cheese hardness had a positive relationship with the values of MNFS, salt in moisture, TN/DM, F/DM, WSN/TN and NPN/TN for samples II and III, while the relationship was inverse for treatments IV and V. Zheng et al (2016) found that reduce in MNFS, F/DM, or storage temperature contributed to an increase in the firmness of sliced cheese like Cheddar, Emmental and Gouda.

The cheese samples contained 1 and 2% DWPP exhibited significant decrease in adhesiveness values while, the cheese samples contained 3 and 4% DWPP presented significant increase after three months of maturation stage. The lowest adhesiveness value was recorded for treatment II (1% DWPP) and the highest value was observed for treatment V (4% DWPP). It is also noticeable from the results of Tables 2 and 5 that the adhesiveness values increased with high level of ripening indices. It was showed that the adhesiveness values of Cheddar cheese increased when the S/M values increased (Chevanan et al. 2006).

Data of Table 5 illustrate the effect of adding DWPP on the cohesiveness of Gouda cheese at the end of ripening period. The cohesiveness values in experimental Gouda cheeses increased significantly (*P* < 0.05) by adding 1 and 2% DWPP. As added DWPP amounts rose from 1 and 2 to 3 and 4%, the cohesiveness significantly (*P* < 0.05) declined which may be attributed to the high moisture content of cheese (Table 2). In general, increasing of cohesiveness levels improves Gouda cheese properties. Yates and Drake (2007) conducted a sensory study on Gouda cheese texture. They concluded that consumers preferred Gouda cheese with a smooth and cohesive texture over one with higher fracturability and firmness.

The springiness values took the same trend of cohesiveness. The springiness levels of Gouda cheese were increased by 14.48, 13.79% for samples II and III respectively clearing significant differences (*P* < 0.05). For treatments IV and V, the springiness levels lowered by 4.14 and 13.56% respectively which also may be due to the high moisture content of cheese. Parra-Ocampo et al (2020) stated that springiness is significantly correlated (*P* < 0.05) with moisture and pH of cheese.

Fortification with 1 and 2% DWPP increased gumminess of Gouda cheese. Adding higher concentrations of DWPP significantly (*P* < 0.05) reduced the cheese gumminess. These findings contradicted with those reported by Henriques et al (2013) who found that hardness and gumminess values of fresh cheese contained liquid whey protein concentrates were similar to control one.

Gouda cheese fortified with 1 and 2% DWPP possessed the greater chewiness values while cheese contained 3 and 4% DWPP had the lowest levels as compared with control. Othman (2008) reported that adding WPC to milk lowered the hardness, cohesiveness, springiness, gumminess and chewiness of low fat soft cheese. However, Henriques et al (2013) found that hardness, chewiness and gumminess values were similar in control and cheese contained whey protein.

### Free fatty acids content (FFA) of Gouda cheese at the end of ripening period

Data of Table 6 illustrate the free fatty acids composition of Gouda cheese after three months of ripening.

**Table 6:** Effect of adding DWPP on free fatty acids (%) content of Gouda Cheese at the end of ripening period

Fatty acids	C	Treatments				
		I	II	III	IV	V
*Saturated fatty acids (SFA) %*						
Butyric	4:0	1.3900	1.0687	0.4473	0.7251	0.1182
Pentanoic	5:0	2.4653	2.5019	2.9449	2.8970	2.8585
Caproic	6:0	1.8958	1.8310	1.8538	1.8899	1.6917
Caprylic	8:0	1.2509	1.2438	1.2346	1.2259	1.2461
Capric	10:0	2.8162	2.7041	2.7718	2.7185	2.6462
Undecanoic	11:0	0.2307	0.2250	0.2309	0.2238	0.2232
Lauric	12:0	3.1387	3.1072	3.0892	3.0313	3.0631
Tridecanoic	13:0	0.3159	0.2450	0.2092	0.2726	0.1746
Myristic	14:0	10.8456	10.8203	10.7482	10.6780	10.6238
Pentadecanoic	15:0	0.6895	0.6645	0.5884	0.5954	0.5986
Palmitic	16:0	30.5617	30.2547	30.2919	29.9507	29.9919
Heptadecanoic	17:0	0.6843	0.5560	0.5494	0.5321	0.5932
Stearic	18:0	12.3246	12.6032	12.5787	12.6349	12.6905
Arachidic	20:0	0.2999	0.2521	0.2309	0.2572	0.2696
Behenic acid	22:0	0.2798	0.2588	0.2266	0.2190	0.1982
	24:0	0.1199	0.0790	0.0952	0.1062	0.0970
Total	69.1088	68.4153	68.0910	67.9576	67.2644	
*Unsaturated fatty acids (USFA) %*						
Myristioleic	14:1	1.0120	1.0060	0.9571	0.9624	0.9617
	15:1	1.0722	1.0551	1.0689	1.0748	1.0548
Palmitioleic	16:1	0.9926	0.9897	0.9781	0.9870	0.9714
	17:1	0.6309	0.7238	0.7850	0.8808	0.9887
Oleic	18:1	24.1675	24.6692	24.9289	24.9975	25.5858
Linoleic	18:2	0.3666	0.3685	0.3780	0.3667	0.3975
α-Linolenic	18:3	2.1822	2.1879	2.1831	2.1832	2.1909
Gadoleic acid	20:1	0.5672	0.5845	0.5989	0.5893	0.5849
Total	30.9912	31.5847	31.8790	32.0417	32.7357	

Treatment I: Gouda cheese (control); Treatment II: Gouda cheese contained 1% DWPP; Treatment III: Gouda cheese contained 2% DWPP; Treatment IV: Gouda cheese contained 3% DWPP; Treatment V: Gouda cheese contained 4% DWPP

### Saturated fatty acids (SFA) content of Gouda cheese

Generally, adding DWPP to cheese lowered SFA values. The content of total saturated fatty acids reduced by 1.00, 1.47, 1.66 and 2.67% with the incorporation of 1, 2, 3 and 4% DWPP to cheese respectively. Nonetheless, not all SFA decreased by DWPP addition, the values of pentanoic (C_5:0_) and stearic (C_18:0_) acids slightly increased in DWPP cheese. In various Gouda cheese treatments, the most abundant SFA was palmitic acid (C_16:0_) followed by stearic (C_18:0_) acid and myristic acid (C_14:0_). Yildiz-Akgul (2018) stated that adding of whey protein isolates (WPI) to yoghurt milk may affect the free fatty acids contents of the resulted yoghurt. The addition of WPI as a technological ingredient to enrich the yoghurt milk with whey proteins led to a remarkable reduction in butyric acid, caproic acid and long-chain fatty acid amounts across the storage period.

### Unsaturated fatty acids (USFA) content of Gouda cheese

As shown in Table 6, pronounced differences between the cheeses samples were observed regarding USFA levels. In contrast to SFA, the values of USFA were higher in cheese fortified with DWPP as compared with control. The amounts of USFA increased by 1.91, 2.86, 3.39 and 5.63% for II, III, IV and V samples respectively. In contrast to most unsaturated fatty acids, myristioleic (C_14:1_) and palmitioleic (C_16:1_) acids decreased in DWPP cheese. Among USFA, oleic acid (C_18:1_) had the highest concentration in all cheese treatments. The role of USFA in the lowering of harmful LDL cholesterol is scientifically established. Nutritionists suggest taking half of oil derived calories from oleic acid (omega-9) to minimize the dangers of cardiovascular diseases (Khan et al. 2018). It is also well known that linoleic acid (C_18:2_—omega-6) and α-linolenic acid (C_18:3_—omega3) have special importance in healthy nutrition. In our study, it was noted that fortification of Gouda cheese with DWPP improved the amounts of oleic acid, linoleic acid (omega-6) and α-linolenic acid especially oleic acid. The levels of oleic acid increased by 2.07, 3.15, 3.43 and 5.87% for samples II, III, IV and V respectively. Mileriene et al (2021) stated that control cheese had higher levels of saturated, short-chain and medium-chain fatty acids, while adding of thermo-coagulated acid whey protein (TAWP) to cheese increased unsaturated, mono- and poli- unsaturated and long-chain fatty acids, Omega3, and Omega6 values. Higher amounts of unsaturated, mono- and poli- unsaturated fatty acids improved thrombogenicity, atherogenicity and hypocholesterolaemic/hypercholesterolaemic indexes in TAWP cheese.

There is an interaction and cross-links between casein matrix and fat phase which can influence textural properties of cheese due to plasticizing impact of fat (Madadlou et al. 2007). As previously mentioned, treatments containing high amounts of DWPP (IV and V) had low hardness compared to other samples. The data of Table 6 indicate that the concentrations of short chain fatty acids (C_8_–C_12_) of samples IV and V were also low. Consequently, the low hardness of cheese is correlated with the low content of short chain fatty acids. In this sense, Aminifar and Emam-Djomeh (2014) cleared that adding of lipase to Lighvan cheese increased short chain fatty acids values and also increased hardness and decreased the brittleness.

From another point of view, unsaturated fatty acids have low melting points. In this investigation, when DWPP was mixed with cheese, the levels of USFA increased, which partially led to a decrease in cheese hardness (treatments IV and V) because of their low melting points comparing with saturated fatty acids. These outcomes are in line with those reported by Hurtaud and Peyraud (2007). They showed butter's texture and hardness are linked to the amounts of SFA and USFA because the lower melting point of USFA forms a less firm. Zoidou et al (2016) stated that texture of traditional whey cheese was softer, less cohesive, gummier, adhesive and chewier as a result of replacement of milk fat with vegetable oil. This was due to the low melting point of vegetable fat and oleic acid in comparison with the high melting point of milk-fat.

## Conclusion

The main purpose of this investigation was to use an inexpensive byproduct to improve the texture of Gouda cheese. Denatured whey protein paste (DWPP) was added to Gouda cheese curd at 1, 2, 3 and 4%. The addition of DWPP to curd cheese affected the physicochemical and rheological properties of Gouda cheese. Values of density and free oil lowered in DWPP samples. Incorporation of 1 and 2% DWPP with cheese increased hardness, cohesiveness, springiness, gumminess and chewiness values whereas adding of higher concentrations decreased them. Levels of saturated fatty acids reduced while unsaturated fatty acid increased in DWPP cheese. Fortification of Gouda cheese with 1 or 2% DWPP improved the texture and healthy properties.

## Data Availability

All data generated or analyzed during this study are included in this published article.

## Abbreviations

**DWPP**: Denatured whey protein paste
**FAMEs**: Fatty acids methyl ester
**Fat/DM**: Fat/Dry Matter
**FFA**: Free fatty acids content
**FO**: Free Oil
**FOFB**: Free Oil on fat basis
**MNFS**: Moisture in nonfat substance
**NPN/TN**: Non protein nitrogen/Total nitrogen
**SFA**: Saturated fatty acids
**TN/DM**: Total nitrogen/Dry Matter
**TPA**: Texture Profile Analysis
**USFA**: Unsaturated fatty acids
**WSN/TN**: Water soluble nitrogen/Total nitrogen
